# Metagenomic Next-generation Sequencing Compared With Blood Culture as First-line Diagnostic Method for Bloodstream Infection in Hematologic Patients With Febrile Neutropenia: A Multicenter, Prospective Study

**DOI:** 10.1093/ofid/ofaf288

**Published:** 2025-05-16

**Authors:** Rui Ma, Yue Yin, Jian-Ping Zhang, Mei-Xiang Zhang, Jing-Rui Zhou, Yun He, Wei Gai, Xiao-Hui Zhang, Yu Wang, Lan-Ping Xu, Kai-Yan Liu, Xiao-Jun Huang, Yu-Qian Sun

**Affiliations:** National Clinical Research Center for Treatment of Hematological Disease, Beijing Key Laboratory of Hematopoietic Stem Cell Transplantation for the Treatment of Hematological Diseases, Peking University People's Hospital, Peking University Institute of Hematology, Beijing, China; Department of Hematology, Peking University First Hospital, Beijing, China; Hebei Yanda Lu Daopei Hospital, Langfang, China; Department of Hematology, Peking University International Hospital, Beijing, China; National Clinical Research Center for Treatment of Hematological Disease, Beijing Key Laboratory of Hematopoietic Stem Cell Transplantation for the Treatment of Hematological Diseases, Peking University People's Hospital, Peking University Institute of Hematology, Beijing, China; National Clinical Research Center for Treatment of Hematological Disease, Beijing Key Laboratory of Hematopoietic Stem Cell Transplantation for the Treatment of Hematological Diseases, Peking University People's Hospital, Peking University Institute of Hematology, Beijing, China; WillingMed Technology (Beijing) Co., Ltd, Beijing, China; National Clinical Research Center for Treatment of Hematological Disease, Beijing Key Laboratory of Hematopoietic Stem Cell Transplantation for the Treatment of Hematological Diseases, Peking University People's Hospital, Peking University Institute of Hematology, Beijing, China; National Clinical Research Center for Treatment of Hematological Disease, Beijing Key Laboratory of Hematopoietic Stem Cell Transplantation for the Treatment of Hematological Diseases, Peking University People's Hospital, Peking University Institute of Hematology, Beijing, China; National Clinical Research Center for Treatment of Hematological Disease, Beijing Key Laboratory of Hematopoietic Stem Cell Transplantation for the Treatment of Hematological Diseases, Peking University People's Hospital, Peking University Institute of Hematology, Beijing, China; National Clinical Research Center for Treatment of Hematological Disease, Beijing Key Laboratory of Hematopoietic Stem Cell Transplantation for the Treatment of Hematological Diseases, Peking University People's Hospital, Peking University Institute of Hematology, Beijing, China; National Clinical Research Center for Treatment of Hematological Disease, Beijing Key Laboratory of Hematopoietic Stem Cell Transplantation for the Treatment of Hematological Diseases, Peking University People's Hospital, Peking University Institute of Hematology, Beijing, China; National Clinical Research Center for Treatment of Hematological Disease, Beijing Key Laboratory of Hematopoietic Stem Cell Transplantation for the Treatment of Hematological Diseases, Peking University People's Hospital, Peking University Institute of Hematology, Beijing, China

**Keywords:** blood culture, bloodstream infection, febrile neutropenia, hematologic diseases, mNGS

## Abstract

Bloodstream infection (BSI) is a frequent but lethal complication in hematologic patients with febrile neutropenia (FN). However, blood culture (BC) only detects an organism in 20%–30% of patients with FN. We aimed to evaluate the diagnostic performance of metagenomic next-generation sequencing (mNGS) as a first-line diagnostic method in BSI. This study was prospectively performed in 4 Chinese hematologic centers. In patients aged ≥15 years with hematologic diseases, peripheral blood specimens were collected per patient for simultaneous BC and mNGS at the onset of FN. The clinical physician and mNGS analysis team were double-blinded, and the adjudication of the clinical diagnosis was evaluated by another expert panel of 4 specialists. The primary endpoint of this study was the diagnostic performance of mNGS. This study was registered on ClinicalTrials.gov. Three hundred FN events were enrolled, including 62 definite BSI, 61 probable BSI, 116 infectious FN other than BSI, 55 noninfectious FN events, and 6 FN of indeterminate cause. Among 62 definite BSI cases, mNGS identified causative pathogens in 59 (95.2%). Concurrent BC initially detected pathogens in 59 cases, and 3 additional pathogens consistent with mNGS were later identified in repeated BC testing. The sensitivity, specificity, positive predictive value, and negative predictive value of mNGS were 95.2%, 94.6%, 95.2%, and 94.6%, respectively. The diagnostic time of mNGS was significantly shorter than that of BC (39.7 ± 15.0 vs 119.8 ± 31.9 hours, *P* < .0001). The findings suggest that the mNGS approach has excellent diagnostic performance for the first-line diagnosis of BSI in patients with FN. The study will promote early diagnosis and better management of the patients.

## Teaser

This multicenter prospective study suggests that mNGS could serve as a promising first-line diagnostic tool for bloodstream infections in febrile neutropenic hematologic patients, demonstrating high sensitivity (95.2%), specificity (94.6%), and significantly shorter turnaround time (39.7 vs 119.8 hours).

Bloodstream infection (BSI) is the most common infection in hematologic patients with febrile neutropenia (FN) [1]. The associated mortality rate is 10%–20% [2] and could be up to 30%–50% if inappropriately managed [1, 3]. However, causative pathogens could be identified in only a few patients with BSI. Blood culture (BC) is the gold standard test for BSI, which is positive in only 20%–30% of patients with FN, and usually requires at least 48–72 hours to identify the pathogen [4–7]. This might delay targeted treatment and increase mortality. Therefore, there is an urgent need for novel diagnostic methods with improved sensitivity, specificity, and rapid turnover.

Metagenomic next-generation sequencing (mNGS) has the advantage of being hypothesis-free and having a wide coverage and faster turnaround time compared with conventional methods [8–10]. Several previous studies have highlighted the promising utility of the mNGS approach in diagnosing BSI compared to BC [11–17]. mNGS approach has demonstrated better sensitivity than BC [14, 18]. However, mNGS is usually the last option for patients with suspected infection who have negative results with traditional methods. Given the fatal consequence of delayed diagnosis in patients with FN, the role of mNGS as a first-line diagnostic method for BSI should be investigated. However, few studies have investigated this role. Two recent prospective studies [6, 18] included only 10 and 14 definite BSI cases; both were single-center studies. Therefore, the clinical significance of mNGS as a first-line diagnostic method for BSI should be further investigated in a well-designed study. Here, we conducted a large-scale, multicenter, and prospective study to comprehensively evaluate the ability of this mNGS approach to identify infectious etiologies of BSI and its potential use as a first-line diagnosis method of BSI in patients with FN.

## METHODS

### Study Design

**Figure 1.**
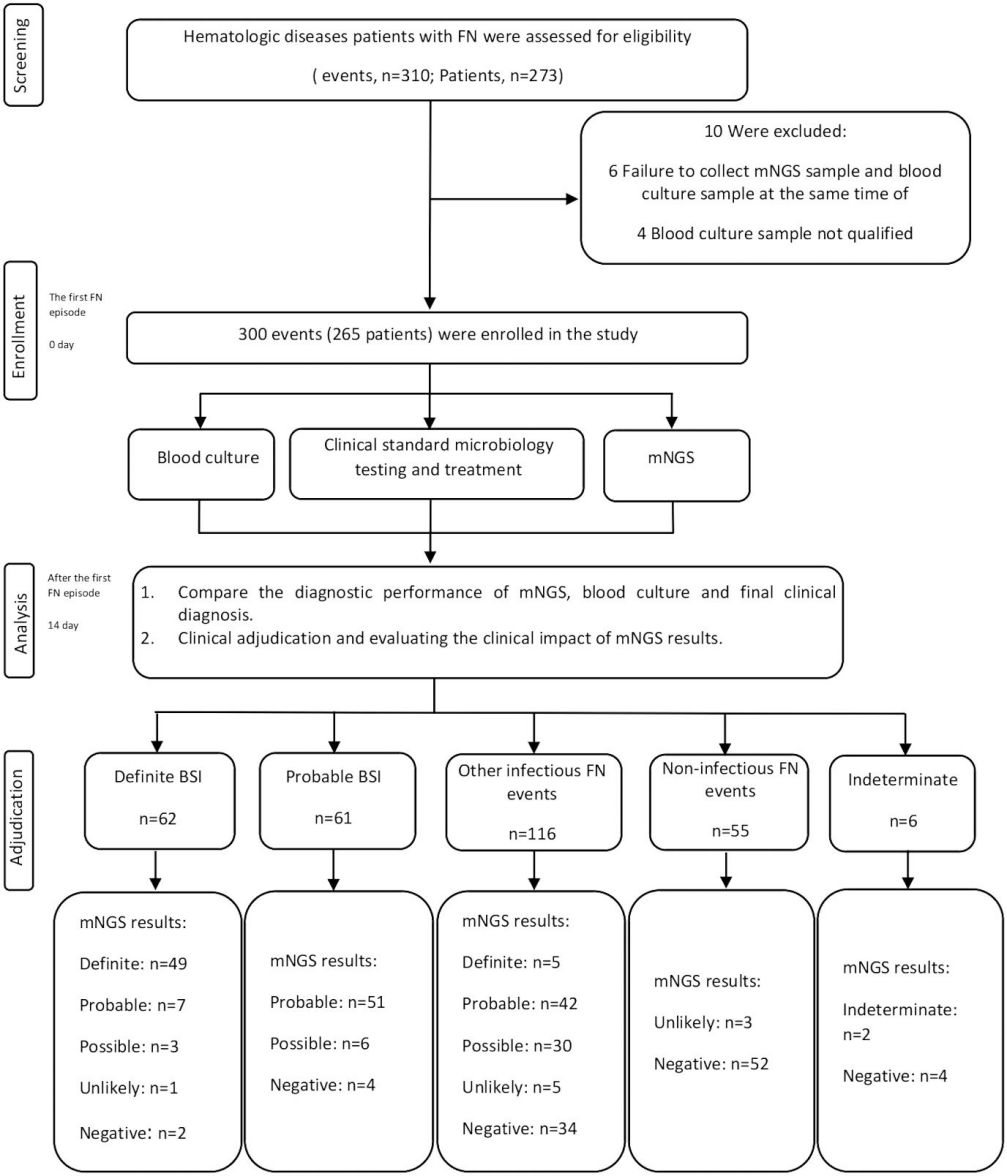
Workflow of the prospective study. BSI, bloodstream infection; FN, febrile neutropenia; mNGS, metagenomics next-generation sequencing.

This multicenter prospective study was performed in 4 Chinese hematologic centers from October 2021 to August 2022. Consecutive patients with hematologic diseases (hematologic malignancies, autologous hematopoietic stem cell transplantation, or allogeneic hematopoietic stem cell therapy [2 days after transplantation]) and neutropenia [absolute neutrophil count <.5 × 10 [9]/L]) were prospectively screened. Inclusion criteria were: (1) age ≥ 15 years and (2) the first FN episode. FN of the same patient with an interval of more than 14 days could be treated as a new enrollment. Patients with active infection or unresolved prior infections were excluded from the study. Prior application of prophylactic antibacterial or antifungal antimicrobials were allowed in enrolled patients. The workflow of the study is presented in Figure 1.

At the onset of FN, peripheral blood specimens were collected from each patient for simultaneous BC and mNGS. Other microbiological samples including serum, respiratory (eg, sputum, bronchoalveolar lavage), urine, cerebrospinal fluid, and tissue biopsies were systematically collected according to treating physician's decisions. The management of FN generally followed the Chinese guidelines [19]. The mNGS results were blinded to the clinical physicians, and all therapeutic interventions were determined independently of the sequencing data during the study period. The clinical diagnosis was blinded to the mNGS analysis team. All patients were followed up for 14 days or until death within 14 days. The study design is summarized as a flowchart in Figure 1. The detailed study protocol is presented in the Supplementary files.

This study was approved by the institutional review board of our hospital. All enrolled patients provided informed consent for participating in the study. The study was registered at http://www.clinicaltrials.gov (No. NCT05149547).

### Study Endpoints

The primary endpoint was the diagnostic performance of mNGS (including sensitivity, specificity, and positive and negative predictive values) in BSI. The secondary endpoints included the diagnostic time of mNGS versus BC and the potential significance of mNGS results for antimicrobial management.

### mNGS

The mNGS process is summarized in Supplementary Figure 1. The methods of sample collection, DNA extraction, library construction, sequencing, data analysis, the pipeline of bioinformatics analyses, and threshold criteria for reporting detected pathogens are described in the Supplementary Method. Microbes were considered potential pathogens if all 3 criteria were met: the reads per 10 million (RPTM) threshold (bacteria/fungi ≥8 RPTM; viruses ≥3 RPTM; *Cryptococcus/Mycobacterium* ≥1 RPTM), the microbes *Z*-score: ≥ 2, and the potential of pathogenicity according to the public literature (described in the Supplementary Method) [8, 11, 20]. All other microbes were considered unlikely or uncertain pathogens.

### Clinical Adjudication

At the end of the follow-up, the final adjudication of the clinical diagnosis was evaluated by an independent expert panel of 4 specialists in hematology as definite BSI, probable BSI, infectious FN events other than BSI, indeterminate, and noninfectious FN (Supplementary Table 1). Definite BSI was defined by concordance between positive BC results and clinical manifestations, with confirmed pathogen causality. Probable BSI was assigned to BC-negative cases with clinical/laboratory evidence strongly implicating bloodstream origin. Infectious FN events other than BSI involved confirmed infections at non-BSI foci (eg, pulmonary, urinary tract) via standard microbiological testing with expert validation. Indeterminate reflected cases with insufficient evidence for definitive classification. Noninfectious FN events were cases adjudicated as non-infectious in origin.

The expert panel also evaluated the possibility of mNGS results as causative pathogens of FN into definite, probable, possible, unlikely, and indeterminate categories. Definite: mNGS was concordant with initial BC or other microbiological results (within 7 days) for at least 1 pathogen, and the pathogen was judged by the expert panel to be a likely cause of BSI in FN. Probable: mNGS was discordant with initial BC, encompassing either discrepancies in detection status (mNGS-positive/BC-negative or vice versa) or mismatches in pathogen identity. According to symptoms, signs, and laboratory findings, the pathogen was judged by the expert panel to be a likely cause of BSI in FN. Possible: mNGS was discordant with initial BC. According to the clinical experience and literature, the pathogen was judged by the expert panel to be a concordant cause of BSI in FN, but it was not a common cause. Unlikely: mNGS result was not a plausible cause of BSI in FN, or the expert panel had a more plausible explanation for BSI in FN. Indeterminate, The expert panel did not have sufficient information to adequately adjudicate and classify the case, and the clinical evidence and microbiological test results did not meet these criteria.

### Definitions

The definition and calculation methods of sensitivity, specificity, positive predictive value, and negative predictive value were based on the previous literature [21]. The diagnostic time was defined as the time from sample collection to when the laboratory report of the final results became available.

### Statistical Methods

#### Sample Size Estimation

This was a diagnostic study based on a comparison of categorical variables (rates). The expected sensitivity and specificity of mNGS, according to the literature, were 92.9% and 62.7% [11], respectively. The 2-sided α value was set at 0.05. The estimated sample size was 258 using the PASS version 15 software (NCSS Statistical Software). The final sample size was 290 events, with a loss rate of 12.5%.

#### Statistical Analyses

Measurement data are expressed as mean ± standard deviation, median, and interquartile range, or proportions (absolute and relative frequencies), as appropriate. Student's *t*-test or Mann–Whitney *U* test was used to compare continuous variables, while the χ^2^ test or Fisher's exact test was used to compare categorical variables. The positive and negative percentage agreements referred to raw concordant percentage. The sensitivity, specificity, positive predictive value, and negative predictive value were evaluated using MedCalc version 14 (MedCalc Software, Mariakerke, Belgium). Statistical analysis was performed using IBM SPSS Statistics version 24 (IBM Corp., Armonk, NY, United States). Differences were considered statistically significant at *P* < .05.

## RESULTS

### Patient Characteristics

During the study period, 310 events (273 hematologic disease patients with FN) were assessed for eligibility. Of these events, 4 were excluded because the BC samples did not meet the quality control requirements, and 6 were excluded because the samples for mNGS and the initial BC were not collected at the same time. Thus, 300 events (265 patients) were enrolled in the final analysis (Figure 1). Basic characteristics of enrolled events and patients are summarized in Table 1.

**Table 1.** Characteristics of Study Participants

Characteristics	No. (%)
Total	300 (100)
Median age, range, y	44 (15–102)
Sex	
Male	145 (55)
Female	120 (45)
Underlying disease	
Acute myeloid leukemia	167 (56)
Acute lymphoblastic leukemia	56 (19)
Myelodysplastic syndrome	15 (5)
Aplastic anemia	6 (2)
Lymphoma	6 (4)
Multiple myeloma	13 (4)
Other diseases	37 (12)
Cause of FN	··
Chemotherapy	192 (64)
Auto-HSCT	23 (8)
Allo-HSCT	85 (28)
Chronic diseases	··
Hypertension	29 (10)
Diabetes	17 (6)
CHD	5 (2)
Hemorrhoids	5 (2)
COPD	4 (1)
FN events classification	··
Definition BSI	62 (21)
Probable BSI	61 (20)
Other FN events	116 (39)
Noninfectious FN events	55 (18)
Indeterminate	6 (2)
Median duration of first pyrexia, range, d	2 (0∼11)
Median time from neutropenia to first pyrexia, range, d	7 (1–29)
14-d mortality	11 (4)

Abbreviations: allo-HSCT, allogeneic hematopoietic stem cell transplantation; auto-HSCT, autologous hematopoietic stem cell transplantation; BSI, bloodstream infection; CHD, congestive heart disease; COPD, chronic obstructive pulmonary disease; FN, febrile neutropenia.

**Figure 2.**
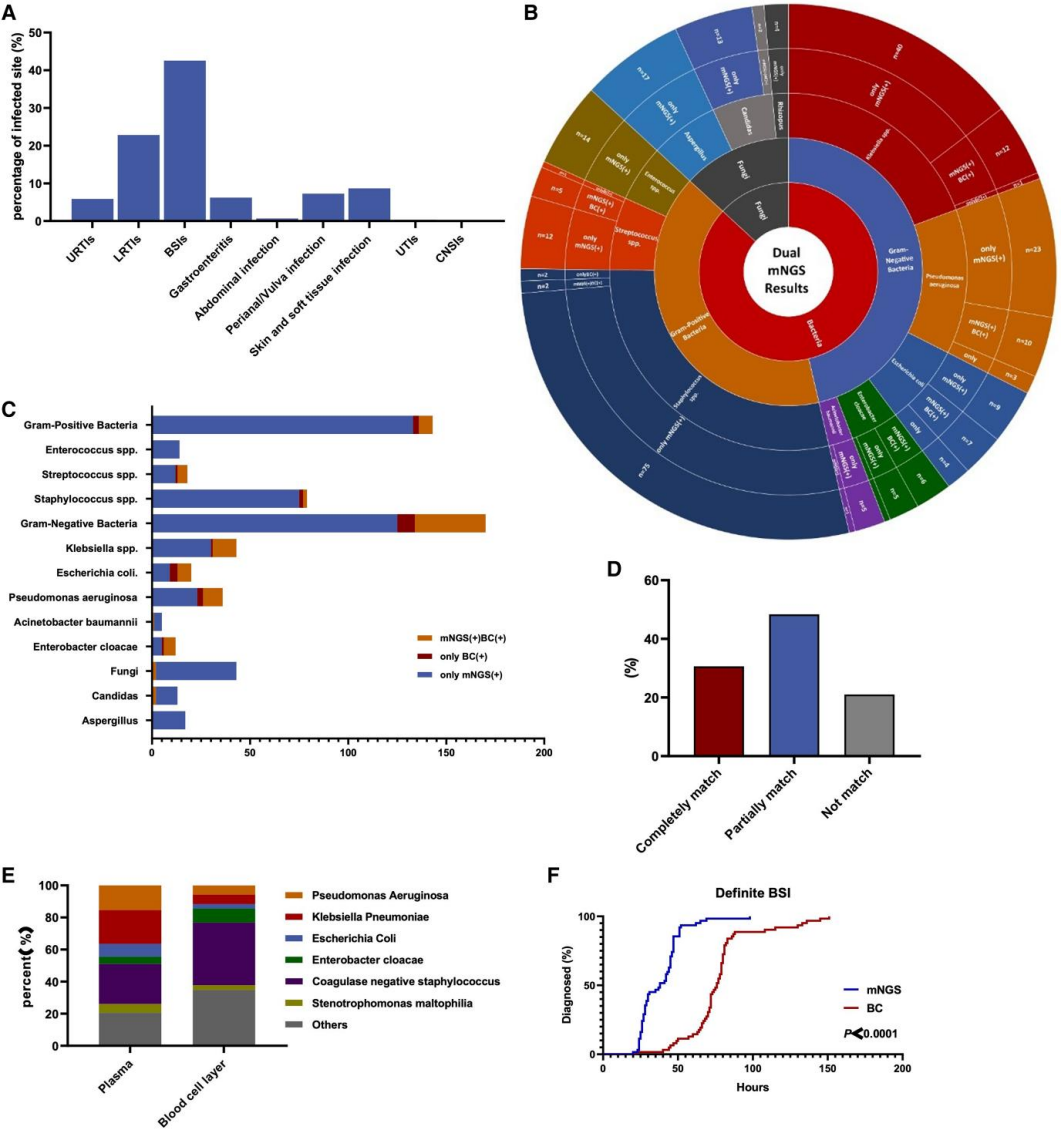
Distribution of microbes, comparison of the detection rates, and distribution of the infection sites involved in this study. *A*, The infection site distribution involved in this study. *B*, Distribution of microbes detected by mNGS in 62 definite BSI events, 61 probable BSI events, and 116 other infectious FN events. *C*, Distribution of microbes detected by mNGS and BC. *D*, Consistency comparison of mNGS and BC in 62 definite BSI events. *E*, Distribution of microbes and comparison of the detection rates between plasma and blood cell layer. *F*, The diagnostic time between mNGS and BC. BC, blood culture; BSI, bloodstream infection; CNSIs, central nervous system infections; FN, febrile neutropenia; LRTIs, lower respiratory tract infections; mNGS, metagenomic next-generation sequencing; URTIs, upper respiratory tract infections; UTIs, urinary tract infection.

Among the 300 FN events, BC was positive in 62 (20.7%) cases. The most frequent pathogens detected in BC were *Pseudomonas aeruginosa* (21%), *Klebsiella pneumoniae* (18%), and *Escherichia coli* (18%). Moreover, 2 cases of fungi were detected by BC, both of which were *Candida tropicalis*. According to clinical adjudication, the 300 FN events were adjudged as follows: 62 definite BSI, 61 probable BSI, 116 infectious FN events other than BSI, 55 noninfectious FN events, and 6 FN events of indeterminate causes. Among the 239 cases of clinically adjudged infectious FN events, BSI was the most common type (123 cases, 42.6%), followed by lower respiratory tract infections (66 cases, 22.8%) and skin or soft-tissue infections (25 cases, 8.7%) (Figure 2*A*).

### Pathogen Profiles Detected by mNGS

Among the 300 FN events, mNGS was positive in 204 (68.0%) cases (except viruses), and 80 of the 204 (39.2%) mNGS results were polymicrobial. The most frequently detected pathogens were *K pneumoniae* (14%), *P aeruginosa* (11%), *Staphylococcus hominis* (6%), *E coli* (5%), and *Staphylococcus haemolyticus* (5%), followed by *Staphylococcus capitis* (4%), *Stenotrophomonas maltophilia* (4%), and *Enterobacter cloacae* (4%) (Supplementary Table 2). Fungi were detected by mNGS in 36 (12%) events: *Aspergillus* spp. was detected in 17 events, *Candida* spp. in 15 events, and *Rhizopus* spp. in 4 events (Figure 2*B*) (Supplementary Table 3). In 2 definite BSI cases, mNGS had detected BSI only in the blood cell layer and had negative results in the plasma, which was consistent with the BC results. The 2 definite pathogens identified were *S pneumoniae* and *S hominis*.

### Diagnostic Performance of mNGS

A comparison of the mNGS and BC results is shown in Figure 2*C*. Most Gram-positive bacteria were detected exclusively by mNGS, with rare co-detection or BC-only positives. Gram-negative bacteria showed higher mNGS-BC concordance and increased BC-only positives. The majority of fungi were identified exclusively by mNGS; only select *Candida* species were co-detected, with virtually no BC-exclusive cases. The positive and negative percentage agreements of mNGS with BC were 79.0% (49/62) and 34.9% (83/238), respectively (Figure 2*D*).

Among the 62 definite BSI cases, mNGS was positive in 60. Among these 60 positive detections, mNGS results were adjudged as true positives in 59 cases (definite causative pathogen in 49 cases, probable causative pathogen in 7 cases, and possible causative pathogen in 3 cases). In 3 BSI cases, mNGS detected *C tropicalis*, *P aeruginosa*, and *K pneumoniae*, whereas the initial concurrent BC was negative. Repeated BC tests detected the same pathogen after 2, 3, and 5 days. mNGS was negative in 3 definite BSI cases. In 2 definite BSI cases, mNGS detection results were negative, while BC results were *Streptococcus gordonii* and *Proteus mirabilis*, respectively. In another definite BSI case, BC was *Staphylococcus epidermidis*, whereas mNGS detected *Propionibacterium humerusii* (finally judged as unlikely to be the causative pathogen of BSI) (Table 2).

**Table 2.** False Negatives and False Positives of mNGS

	Adjudication of FN Events^a,b^	mNGS Results	Adjudication of mNGS Results	BC Results
False negatives	Definite BSI (n = 3)	*Propionibacterium humerusii*	Unlikely	*Staphylococcus epidermidis*
		Negative	··	*Streptococcus gordonii*
		Negative	··	*Proteus mirabilis*
False positives	Noninfectious events (n = 3)	*Helicobacter pylori*	Unlikely	Negative
		*H pylori*	Unlikely	Negative
		*Staphylococcus capitis; Aeromonas hydrophila*	Unlikely	Negative

Abbreviation: mNGS, metagenomics next-generation sequencing.

^a^The effectiveness was judged according to the literature on antimicrobial susceptibility of the microorganism under consideration.

^b^The final adjudication of the clinical diagnosis was evaluated by an independent expert panel of four specialists in hematology.

Among the 61 probable BSI cases, mNGS was positive in 57 (93.4%). The mNGS results were adjudged as probable causative pathogens in 51 cases, possible causative pathogens in 6 cases, and negative in 4 cases. The pathogen detected by mNGS had a similar distribution in definite BSI and probable BSI (Supplementary Figure 2).

Among the 55 noninfectious FN cases, mNGS was positive in 3 cases. Among these 3 cases, 1 showed *S capitis* and *Aeromonas hydrophila*, and the other 2 cases showed *Helicobacter pylori* (Table 2).

Among the 49 cases in which mNGS was consistent with BC, mNGS detected multiple microbes in 19 (38.8%) cases. According to the predominant rule, the predominant pathogen was consistent with the BC results in 16 of 19 cases (84.2%). In the 3 inconsistent cases, the BC results were *S pneumoniae, Aeromonas caviae*, and *E coli*. The predominant mNGS pathogens were *S capitis*, *Staphylococcus xylosus*, and *Staphylococcus equorum* (Supplementary Table 4).

The sensitivity, specificity, positive predictive value, and negative predictive value of mNGS (blood cell layer + plasma) were 95.2%, 94.6%, 95.2%, and 94.6%, respectively. The sensitivity of mNGS when testing blood cell layer alone and plasma alone was 38.7% and 90.3%, with corresponding specificity values of 100% and 94.6%, respectively (Table 3). Distribution of microbes and comparison of the detection rates between the plasma and blood cell layer summarized in Figure 2*E*.

**Table 3.** Diagnostic Performance of mNGS

		mNGS Positive^a^	mNGS Negative	Sensitivity% (95% CI)	Specificity% (95% CI)	Positive Predictive Value% (95% CI)	Negative Predictive Value% (95% CI)
mNGS using whole blood (N = 300)	Definite BSI^b^	59	3	95.2 (86.5–99.0)	94.6 (84.9–98.9)	95.2 (86.5–99.0)	94.6 (84.9–98.9)
	Noninfectious FN	3	52				
mNGS using blood cell (N = 300)	Definite BSI^b^	24	38	38.7 (26.6–51.9)	100.0 (93.5–100.0)	100.0 (85.7–100.0)	59.1 (48.5–69.2)
	Noninfectious FN	0	55				
mNGS using plasma (N = 300)	Definite BSI^b^	56	6	90.3 (80.1–96.4)	94.6 (84.9–98.7)	94.9 (85.6–98.9)	89.7 (78.8–96.1)
	Noninfectious FN	3	52				

Abbreviations: BC, blood culture; BSI, bloodstream infection; CI, confidence interval; FN, febrile neutropenia; mNGS, metagenomics next-generation sequencing.

^a^Definite, probable, and possible cases were regarded as true positives of the mNGS results.

^b^Blood culture proved BSI.

### mNGS Has Shorter Diagnostic Time Than BC

Compared with BC, the diagnostic time of mNGS was significantly shorter (39.7 ± 15.0 vs 119.8 ± 31.9 hours, *P* < .0001) (Figure 2*F*). Among the definite BSI events, the real-time availability of mNGS results would have provided an earlier result in 91.9% and facilitated an earlier diagnosis in 91.8% of the definite cases with positive BC (n = 45). The panel evaluated the efficacy of empirical antimicrobial therapy on organisms detected by mNGS. During the initial treatment phase (0–48 hours), empirical antimicrobial regimens were deemed definitely effective, probably effective, likely effective, and not effective in 19.0% (57/300), 24.7% (74/300), 6.3% (19/300), and 15.3% (46/300) of cases, respectively. For follow-up treatment (after 48 hours), these proportions shifted to 22.0% (66/300), 25.7% (77/300), 5.7% (17/300), and 12.0% (36/300). The real-time availability of mNGS results could have led to changes in antimicrobial agents in 52.3% of cases, where 29.7% (89/300) required expanded coverage for pathogens not initially targeted, 2.0% (6/300) necessitated escalation, and 20.6% (62/300) allowed de-escalation or discontinuation of current therapies, including termination of 13% coverage of methicillin-resistant *M aureus* (Supplementary Table 5).

## DISCUSSION

To the best of our knowledge, our study is the first multicenter prospective study to evaluate the diagnostic performance of mNGS as a first-line diagnostic method for BSI among hematologic patients with FN. In this large prospective multicenter study, we demonstrated that the mNGS approach has an excellent performance in the first-line diagnosis of BSI, with a sensitivity and specificity of 95.2% and 94.6%, respectively.

The excellent diagnostic performance of mNGS in the current study may be greatly attributed to the improved method. First, the detection of only plasma cell-free DNA (cfDNA) might miss the pathogens in cells. However, the significant host DNA contamination limits the use of whole blood for mNGS [22]. With recent advances in the host DNA depletion technique [23–25], detection from both the plasma and blood cell layer might have increased the sensitivity of mNGS compared with the mNGS approach using only plasma cfDNA. In the current study, we used an mNGS approach detecting both plasma cfDNA and blood cell layer. This approach has the potential for better sensitivity and specificity. Notably, 2 cases were detected only in the blood cell layer, which were definite BSI. Second, cfDNA comes from not only viable pathogens in the blood but also microorganisms at other body sites, whereas pathogens detected in blood corpuscles might suggest the site of BSI [26, 27]. Our study demonstrated that the specificity of the blood cell layer mNGS (100%) was the same to that of BC (100%). Third, several strategies were adopted to decrease the false positives: (1) negative control using patients with asymptomatic neutropenia and (2) strict bioinformatics rules; only those fulfilling all 3 rules were considered positive results. We observed three false positives (*H pylori, S capitis, A hydrophila*, all adjudicated as unlikely pathogens by the expert panel) in noninfectious events, which may be due to contamination, commensal organisms, or limitations in clinical adjudication, underscoring the necessity of incorporating mNGS results within the clinical and laboratory framework to mitigate overinterpretation of incidental microbial signals. Nevertheless, despite of the remaining challenge of false positivity in the clinical interpretation, the mNGS approach combining detection on plasma and blood cells may further enhance diagnostic efficiency in patients with FN. However, due to the significantly increased cost of this approach, clinical implementation requires careful consideration, particularly in resource-limited settings where cost-effectiveness must be balanced against diagnostic benefits.

Differences in detection rates were observed between mNGS and BC across Gram-positive bacteria, Gram-negative bacteria, and fungi, which can be attributed to methodological distinctions, pathogen-specific biological characteristics, and clinical contexts. mNGS demonstrated superior sensitivity compared to BC for Gram-positive bacteria, which aligns with the known limitations of BC for detecting fastidious or slow-growing organisms. Additionally, mNGS can identify fragmented DNA from nonviable or antibiotic-exposed pathogens, particularly relevant in immunocompromised patients receiving prophylactic antimicrobials [11]. In contrast, Gram-negative bacteria showed higher concordance between mNGS and BC, with BC exhibiting increased positivity rates, likely reflecting the rapid proliferation of Gram-negative pathogens in aerobic blood culture, coupled with their higher circulating biomass in bloodstream infections. However, mNGS adds value in identifying polymicrobial infections or resistance markers (eg, ESBL genes) [12]. BC's limitations in fungal detection are well-documented, as fungal cultures require prolonged incubation and specialized media, whereas mNGS identifies fungal DNA irrespective of viability [6]. These findings emphasize the complementary roles of mNGS and BC. Although mNGS excels in detecting fastidious, low-biomass, or non-culturable pathogens, BC remains indispensable for antimicrobial susceptibility testing and confirming viable infections.

The detection of multiple microbes is common. These polymicrobial cases illustrate the complexity of clinical presentations in immunocompromised hosts that are often co-infected with multiple organisms. Oral, skin, and gut florae were commonly identified as concurrent pathogens [28–30]. The frequent detection of multiple microbes by mNGS may lead to antimicrobial overuse in real-world practice, although in the present study, the mNGS result was blinded to the treating clinicians. Currently, there is no consensus regarding the identification of the major causative pathogen. Quantitative measurements of organism predominance may provide a better understanding of the significance of polymicrobial detection. In a report of 167 asymptomatic patients undergoing microbial cfDNA sequencing, mNGS detected low-level (mostly apathogenic human commensals) organisms in 23% of patients, whereas true infections displayed high-level concentrations [11]. In the current study, we adopted a “predominant rule,” which has excellent agreement with BC, emphasizing the necessity of integrating laboratory results with traditional microbiology frameworks and clinical adjudication.

There remained 3 false-negative cases of definite BSI. Possible explanations include the following: (1) the results of BC in these 3 patients might be false positive since the pathogens are common contaminants (*Streptococcus gordonii*, *P mirabilis*, and *P humerusii*) and (2) the low amount of pathogen DNA might be another possible reason. When patients have no infection sites, the negative results of mNGS could preclude infection in most situations. Therefore, it is important to combine the results of mNGS with clinical situations. In addition, the host immune biomarker may improve the diagnostic yield [31, 32].

Despite the advances, our study has some limitations that should be acknowledged. First, prophylactic antibiotics used in FN patients may have reduced BC sensitivity despite resin-based mitigation. Thus, reliance on BC as the gold standard comparator may underestimate mNGS's diagnostic yield in culture-negative cases. Second, although standardized operating procedures were stringently implemented, the absence of formal inter-center and inter-rater comparative analysis may obscure potential site-specific variations, warrants consideration in future multicenter studies.

In conclusion, our findings suggest that the mNGS approach has excellent diagnostic performance for the first-line diagnosis of BSI in patients with FN with hematologic disorders. However, the significance of first-line mNGS results-based interventional strategy should be further investigated in the future.

## Supplementary Material

ofaf288_Supplementary_Data

## Supplementary Data

Supplementary materials are available at *Open Forum Infectious Diseases* online. Consisting of data provided by the authors to benefit the reader, the posted materials are not copyedited and are the sole responsibility of the authors, so questions or comments should be addressed to the corresponding author.
