# Supplementary material for: Metagenomic Next-generation Sequencing Compared With Blood Culture as First-line Diagnostic Method for Bloodstream Infection in Hematologic Patients With Febrile Neutropenia: A Multicenter, Prospective Study
Source: Open Forum Infect Dis. 2025 May 16;12(6):ofaf288. doi: 10.1093/ofid/ofaf288 (PMC12125677; doi:10.1093/ofid/ofaf288)
Supplement: ofaf288_Supplementary_Data [file ofaf288_supplementary_data.zip › Supplementary figures (1).docx]

**Supplementary figures**


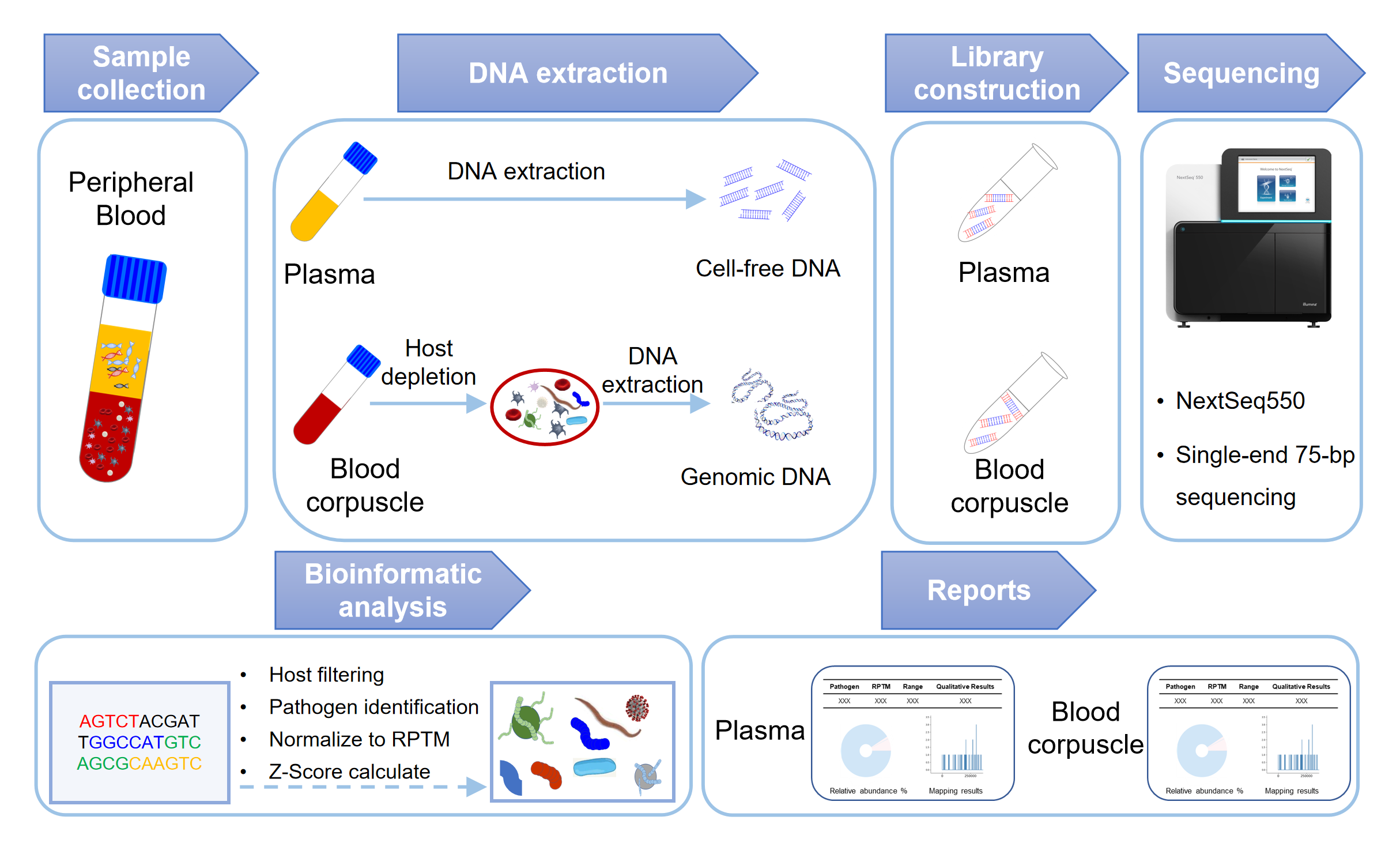


**Figure S1** Metagenomic next-generation sequencing (mNGS) detection workflow.

**Figure S2.** Distribution of microbes detected by mNGS in 62 definite BSI events, 61 probable BSI events and 116 other infectious FN events.
