# Supplementary material for: Metagenomic Next-generation Sequencing Compared With Blood Culture as First-line Diagnostic Method for Bloodstream Infection in Hematologic Patients With Febrile Neutropenia: A Multicenter, Prospective Study
Source: Open Forum Infect Dis. 2025 May 16;12(6):ofaf288. doi: 10.1093/ofid/ofaf288 (PMC12125677; doi:10.1093/ofid/ofaf288)
Supplement: ofaf288_Supplementary_Data [file ofaf288_supplementary_data.zip › Supplementary Method-revised.docx]

**Supplementary Method**

**Blood culture**

***1. Sample collection***

Blood culture samples were collected concomitantly with mNGS samples for patients with febrile neutropenia (FN). For each patient, four blood samples (10 mL per blood culture bottle) were obtained via bilateral antecubital fossa venipuncture using sterile technique. In patients with a peripherally inserted central catheter (PICC), paired specimens were collected: two from the PICC lumen and two from peripheral veins of the contralateral arm. Each collection site provided one aerobic and one anaerobic culture bottle to optimize microbial recovery. Blood culture bottles containing adsorbent polymeric resins were used across all participating centers to minimize influence of antibiotics present in blood samples collected.

***2. Incubation and reporting protocol***

The collected culture bottles were transferred to laboratory within two hours of collection and incubated in 35-37 °C for a standard 5-day period. Prolonged incubation was required for some slow-growing fastidious bacteria (e.g., *Bartonella*, *Listeria* spp., and *Nocardia* spp.) and biphasic fungi. Automated blood culture systems periodically detected CO₂ concentration or pressure changes generated by microbial metabolic activity through electronic sensors. Upon a positive blood culture, a smear and Gram staining was performed immediately, and the results including type and number of positive blood culture bottles, time of alert, Gram staining characteristics and morphology of the microorganisms was reported to the clinician within 1 hour. The positive culture broth was subcultured onto appropriate media, and direct antimicrobial susceptibility testing (AST) was performed based on smear results. The final report included the species identification, time to positivity (in hours), and standardized AST results. A final report would also be issued if the culture remains negative after 5 days of culture.

***3. Contamination Interpretation Criteria***

Commensal skin flora (e.g., *Staphylococcus epidermidis*, *Cutibacterium acnes*, *Clostridium* spp., *diphtheroids*) and environmental organisms (e.g., *Acinetobacter* spp., *Bacillus* spp.) were presumptively considered as contaminants unless meeting clinical significance criteria:

a) The same organism was isolated from blood cultures collected from different sites;
b) The same organism was repeatedly isolated with identical antimicrobial susceptibility profiles.

Contaminants should be reported to clinicians with a note indicating possible contamination (e.g., *Cutibacterium* *acnes*, a skin commensal).

This blood culture protocol aligns with *Operating procedures of blood culture for clinical microbiology laboratory (WS/T 503-2017)* guidelines in China.

**Metagenomic next-generation sequencing**

***1. Sample collection and DNA extraction***

Whole blood samples (2-5 mL) were collected in anticoagulation tubes and transported at 4℃ after collection. After centrifugation at 1900 g for 10 minutes at 4℃, plasma and blood corpuscle were separately collected for further detection.

Cell-free DNA was extracted from plasma using PathoXtract® cell-free Nucleic Acid Kit (WYXM03010S, Willingmed Corp, Beijing, China).

For blood corpuscle, DNA was extracted using PathoXtract® HemoCyte whole Blood Pathogen DNA Enrichment Kit (WYXM03210S, Willingmed Corp, Beijing, China). The first step is to selectively lysis the blood cells by a saponin solution, so the nucleic acids from human cells are released in the solution. Then a nuclease is added in the solution to digest the nucleic acids to fragments shorter than 20 bp, which cannot be recycled during nucleic acid extraction process. At last, the solution is centrifugated and the supernatant is removed to enrich pathogens. The pellets are used to extract nucleic acid, library construction and sequencing.

DNA was eluted with 50 μL of nuclease-free water.

***2. Library construction, sequencing, and data analysis***

Libraries for mNGS were prepared from cfDNA using the KAPA DNA HyperPrep Kit (KK8504, KAPA, Kapa Biosystems, Wilmington, MA, United States) and genomic DNA using the Illumina® DNA Prep, (M) Tagmentation (20018705, Illumina) according to the manufacturer’s protocol. Sequencing of the libraries was performed on NextSeq™ 550Dx (Illumina) and 20 million sequencing reads were acquired for each sample. The non-template controls (NTCs) of double distilled water were processed in parallel with patient samples in each test to exclude environmental and laboratory contaminants.

***3. Pipeline of bioinformatics analysis***

The genomic data of bacteria, fungi, viruses, parasites, archaea, and other pathogenic microorganisms were obtained from NCBI GenBank, and the clinical application level reference database of pathogenic microorganisms was constructed through genomic filtering, screening, and validation. Sequencing data were processed automatically to get the detection report. The detail pipeline for data analysis were as follows: After obtaining the FASTQ format data, low-quality or undetected sequences, contaminated sequences by splices, high coverage repeats, and short read length sequences were filtered to retain high-quality sequencing data. The high-quality sequencing data were compared with the human reference genome GRCH37 (hg19) by alignment software to remove the human host sequence. Then the obtained clean data were aligned with the established reference database of pathogenic microorganisms to complete the annotation of pathogenic microorganism species information and give the final analysis and identification results of microorganisms.

***4. Threshold criteria for reporting detected pathogens***

The mapped reads number of each microbe in each sample was screened in three ways to avoid over-interpretation of the sequencing data. Microbes were considered potential pathogens if all the three criteria described below were met (Figure 1). All other microbes were considered unlikely or uncertain pathogens.

(1) the reads per ten million (RPTM) value

For identification of pathogen, a RPTM value was used to identify positive pathogens, which defined as detected number of pathogen specific reads per 10 million. The RPTM ratio metric (RPTM-r), defined as RPTM _Sample_/RPTM_NTC_, set to 1 in NTC samples when the microbial taxonomic value of a given species or genus is less than 1. Thus, Bacteria (except Mycobacteria), fungi (except Cryptococcus), Mycoplasma, Chlamydia, and parasites with RPTM ≥ 8, viruses with RPTM ≥ 3, special pathogens (including Cryptococcus and Mycobacterium) with RPTM_sample_ ≥ 1, was identified as positive. For common colonized bacteria and contaminating microorganisms, such as *Prevotella pallidum*, *Treponema maltophilia*, *Neisseria subflava, Rothia dentocariosa*, etc., were considered negative in this study.

(2) the microbes Z-score

Z-score was used to show the abundance of mapped reads relative to the same microbe at the species level in the samples of this study cohort. Potential pathogens were defined as those with Z-score≥2.


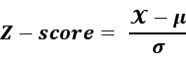


(χ is the log10 transformation of RPTM of one microbe; µ is the mean of log 10 RPTM of the same microbe in this study cohort; σ is the SD of log 10 RPTM of the same microbe in this study cohort.)

(3) the microbe was known to be potentially pathogenic

The potentially pathogen that have been reported in the published papers and expert statements were reserved. Some pathogenic microbes that known as non-pathogenic or without disease-related research were identified as negative.

***5. Negative Control***

Additional context was provided by assessing test performance on 16 asymptomatic neutropenia patients. Of these 16 asymptomatic samples, 50% had no microorganisms reported. Among the 50% of samples in which microbial result was reported, 4 samples detected single species, 3 samples detected double species, and 1 sample detected 6 species. Pathogens detected in these samples were regarded as background pathogens and were not regarded as pathogens in the mNGS report. These pathogens are commonly present as human commensals. In general, the RPTM of mNGS detected from asymptomatic samples were lower than the of confirmed pathogens and similar to the unlikely pathogens **(Figure 2 and Table 1)**.


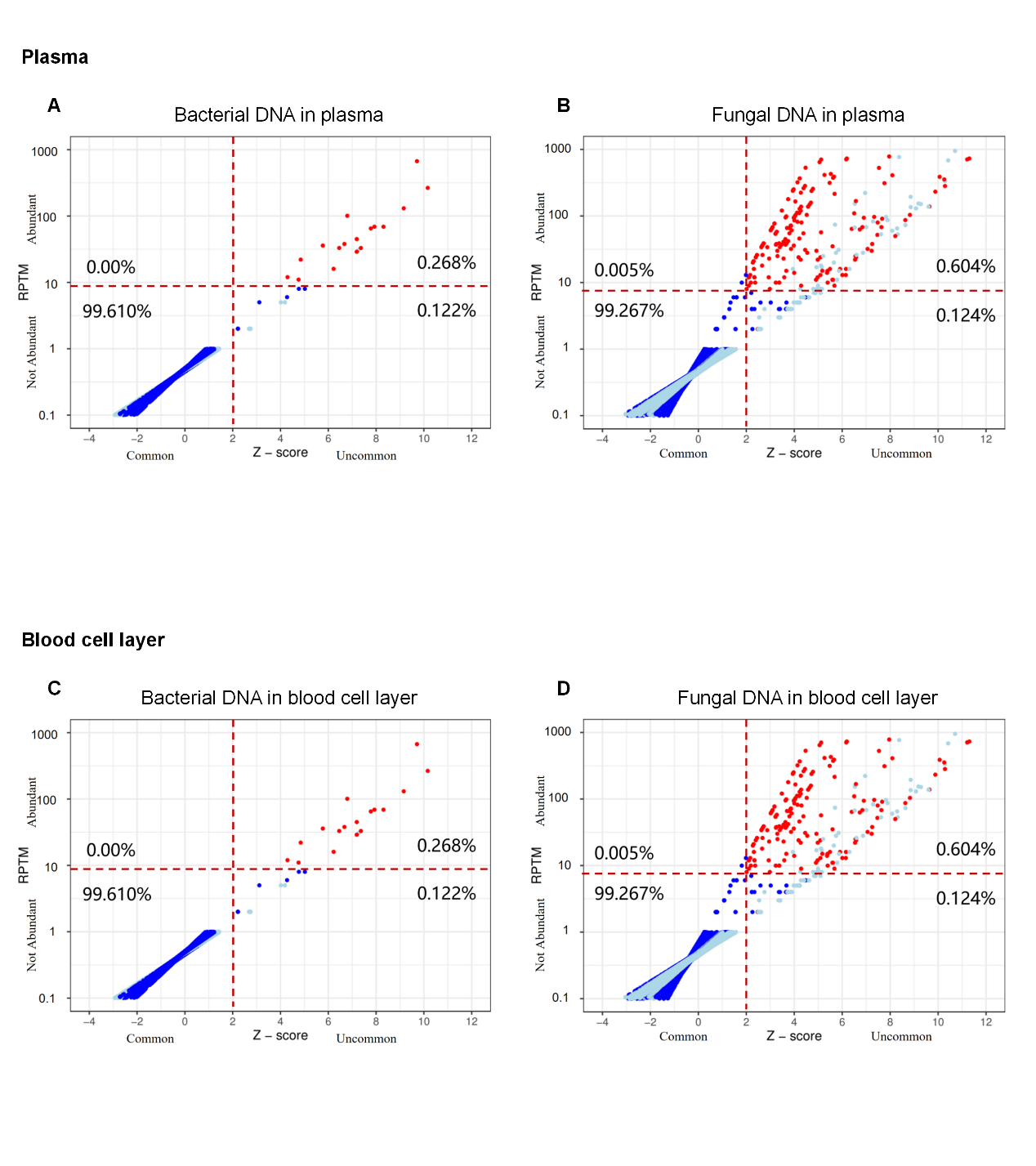


**Figure 1. Microbial alignments detected in the plasma and blood cell layer of FN patients.**

Red dots represent potentially pathogenic microbes that are both abundant (≥8 rpm for bacteria and fungi ) and identified at levels greater than most other samples in the cohort (Z-score≥2). Blue dots represent all other potentially pathogenic microbes; light blue dots represent typically nonpathogenic microbes. (A) bacterial DNA in plasma, (B) fungal DNA in plasma, (C) bacterial DNA in blood cell layer, and (D)fungal DNA in blood cell layer.

**
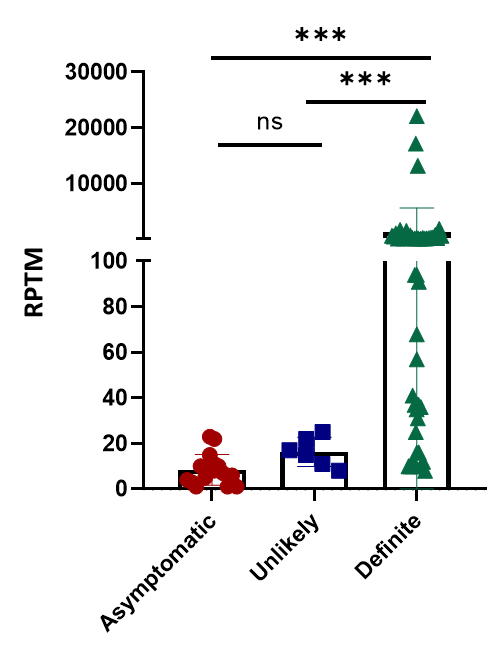
**

**Figure 2. The RPTM of mNGS detected from asymptomatic samples (n= 16) and compared with definite pathogens (n= 49) and unlikely pathogens (n=6).**

✱✱✱,*P*＜0.001; ns, not significant difference.

| **No.** | **Organisms** | | | | | |
| --- | --- | --- | --- | --- | --- | --- |
| 1 | *Prevotella oralis* | *Tannerella forsythia* | *Weissella confusa* | *Streptococcus vestibularis* | *Rothia dentocariosa* | *Fusobacterium nucleatum* |
| 2 | *Acinetobacter radioresistens* | *Acinetobacter johnsonii* | N | N | N | N |
| 3 | [*Psychrophilic pseudomonads*](https://www.baidu.com/link?url=73FWR-g1qbfx_qYYLdjXA4Oxa1lQhjhqFossbHxwRMjz2-w8dspr-zRPUXnEBNVJj5mZpisZe1tdSv2-Iw5QtsDWcD_K20Esa11BNgF6JNpQ7LjSEJickeUbT7a-Hx42&wd=&eqid=8a6f0f4c004199fc0000000263f10930) | *Corynebacterium parvum* | N | N | N | N |
| 4 | *Acinetobacter calcoaceticus* | *Corynebacterium striatum* | N | N | N | N |
| 5 | *Corynebacterium coyleae* | N | N | N | N | N |
| 6 | *Gordona bronchialis* | N | N | N | N | N |
| 7 | *Streptococcus*  *Salivarius* | N | N | N | N | N |
| 8 | *Corynebacterium tuberculostearicum* | N | N | N | N | N |
| 9 | N | N | N | N | N | N |
| 10 | N | N | N | N | N | N |
| 11 | N | N | N | N | N | N |
| 12 | N | N | N | N | N | N |
| 13 | N | N | N | N | N | N |
| 14 | N | N | N | N | N | N |
| 15 | N | N | N | N | N | N |
| 16 | N | N | N | N | N | N |

**Table 1. Organisms detected by dual mNGS in 16 asymptomatic FN patients**
