# Supplementary material for: Metagenomic Next-generation Sequencing Compared With Blood Culture as First-line Diagnostic Method for Bloodstream Infection in Hematologic Patients With Febrile Neutropenia: A Multicenter, Prospective Study
Source: Open Forum Infect Dis. 2025 May 16;12(6):ofaf288. doi: 10.1093/ofid/ofaf288 (PMC12125677; doi:10.1093/ofid/ofaf288)
Supplement: ofaf288_Supplementary_Data [file ofaf288_supplementary_data.zip › Supplementary Tables-revised.docx]

**Supplementary tables**

**Supplementary Table S1.** **Definition of clinical adjudication for FN events**

| **FN events** | Definite BSI^b^ | BC was positive and clinical presentation was concordant with at least one pathogenic infection identified in the blood culture, which was judged by the expert panel to be a likely cause of BSI in FN. |
| --- | --- | --- |
|  | Probable BSI | BC was negative. According to symptoms, signs and laboratory findings, blood was judged by the expert panel to be a likely site of FN. |
|  | Other infectious FN events | BC was negative. Infection foci in other sites had been detected by SMT. According to symptoms, signs and laboratory findings, the infection foci was judged by the expert panel to be a likely cause of FN. |
|  | Indeterminate | The expert panel did not have sufficient information to adequately adjudicate and classify the case, and the clinical evidence and microbiological test results did not meet the above criteria. |
|  | Non-infectious FN events | The expert panel adjudicated it a non-infectious event. |
| Abbreviation: mNGS, metagenomics next-generation sequencing; BSI, bloodstream infection; BC, blood culture; FN, febrile neutropenia; SMT, standard microbiological test.  ^a^ mNGS results were limited to bacteria, fungi, parasites and no viruses were considered.  ^b^ Clinical adjudication BSI events included definite BSI and probable BSI. | | |

**Supplementary Table S2. List of potentially pathogenic bacteria in the plasma and blood cell layer of FN patients**

|  | Bacterial species | Detection frequency |
| --- | --- | --- |
| Plasma | *Klebsiella pneumoniae* | 25 |
|  | *Pseudomonas aeruginosa* | 19 |
|  | *Escherichia coli* | 17 |
|  | *Staphylococcus hominis* | 15 |
|  | *Stenotrophomonas maltophilia* | 13 |
|  | *Staphylococcus capitis* | 11 |
|  | *Staphylococcus epidermidis* | 9 |
|  | *Enterobacter cloacae* | 8 |
|  | *Staphylococcus haemolyticus* | 7 |
|  | *Staphylococcus cohni* | 5 |
|  | *Acinetobacter baumannii* | 5 |
|  | *Helicobacter pylori* | 3 |
|  | *Staphylococcus warneri* | 3 |
|  | *Aeromonas hydrophila* | 3 |
|  | *Serratia marcescens* | 3 |
|  | *Enterococcus faecium* | 2 |
|  | *Streptococcus pneumoniae* | 2 |
|  | *Bacteroides fragilis* | 2 |
|  | *Moraxella osloensis* | 2 |
|  | *Gardnerella vaginalis* | 1 |
|  | *Burkholderia cepacia* | 1 |
|  | *Burkholderia cepacia* | 1 |
|  | *Porphyromonas gingivalis* | 1 |
|  | *Corynebacterium acidulatum* | 1 |
|  | *Streptococcus lactis* | 1 |
|  | *Proteus mirabilis* | 1 |
|  | *Ralstonia pickettii* | 1 |
|  | *Entercoccus avium* | 1 |
|  | *Enterococcus raffinosus* | 1 |
|  | *Klebsiella michigan* | 1 |
|  | *Mycobacterium cosmeticum* | 1 |
|  | *Lactobacillus crispatus* | 1 |
|  | *Ralstonia mannitolilytica* | 1 |
|  | *Mycobacterium tuberculosis* | 1 |
|  | *Streptococcus mitis* | 1 |
|  | *Staphylococcus lentus* | 1 |
|  | *Tannerella forsythensis* | 1 |
|  | *Leuconostoc mesenteroide* | 1 |
|  | *Corynebacterium propinquum* | 1 |
|  | *Elizabethkingia anophelis* | 1 |
|  | *Staphylococcus aureus* | 1 |
|  | *Mycobacterium gallinarum* | 1 |
| Blood cell layer | *Staphylococcus hominis* | 9 |
|  | *Staphylococcus haemolyticus* | 6 |
|  | *Klebsiella pneumoniae* | 5 |
|  | *Staphylococcus capitis* | 4 |
|  | *Enterobacter cloacae* | 4 |
|  | *Staphylococcus epidermidis* | 4 |
|  | *Roseomonas mucosa* | 3 |
|  | *Staphylococcus cohni* | 3 |
|  | *Pseudomonas aeruginosa* | 2 |
|  | *Acrococcus uiridans* | 2 |
|  | *staphylococcus saprophyticus* | 2 |
|  | *Moraxella osloensis* | 2 |
|  | *Acinetobacter ursingii* | 1 |
|  | *Staphylococcus warneri* | 1 |
|  | *Streptococcus dysgalactiae* | 1 |
|  | *Stenotrophomonas maltophilia* | 1 |
|  | *Dermatococcus* | 1 |
|  | *Ochrobactrum anthropi* | 1 |
|  | *Acinetobacter junii* | 1 |
|  | *Enterococcus casseliflavus* | 1 |
|  | *Lautropia mirabilis* | 1 |
|  | *Staphylococcus xylosus* | 1 |
|  | *Staphylococcus equorum* | 1 |
|  | *Corynebacterium jeikeium* | 1 |
|  | *Streptococcus pneumoniae* | 1 |
|  | *Moraxella nonliquefaciens* | 1 |
|  | *Escherichia coli* | 1 |
|  | *Pantoea agglomerans* | 1 |

**Supplementary Table S3.** **List of potentially pathogenic fungi in the plasma and blood cell layer of FN patients**

|  | Fungi species | Detection frequency |
| --- | --- | --- |
| Plasma | *Aspergillus fumigatus* | 5 |
|  | *Aspergillus flavus* | 5 |
|  | *Candida tropicalis* | 3 |
|  | *Candida sake* | 3 |
|  | *Rhizomucor pusillus* | 3 |
|  | *Rhizopus microsporus* | 2 |
|  | *Kodamaea ohmeri* | 1 |
|  | *Aspergillus griseus* | 1 |
|  | *Cunninghamella bertholletiae* | 1 |
|  | *Candida parapsilosis* | 1 |
|  | *Aspergillus sydowii* | 1 |
|  | *Fusarium verticillioides* | 1 |
|  | *Aspergillus versicolor* | 3 |
| Blood cell layer | *Candida tropicalis* | 3 |
|  | *Aspergillus niger* | 3 |
|  | *Candida glabrata* | 2 |
|  | *Aureobacidium pullulans* | 1 |
|  | *Rhizopus oryzae* | 1 |
|  | *Exophiala dermatitidis* | 1 |
|  | *Candida tropicalis* | 1 |
|  | *Candida famata* | 1 |

**Supplementary Table S4. Organisms identified by dual mNGS in 62 definite BSI events**

| **No.** | **Blood culture results**  **(n=62) ^a^** | **Clinical adjudication of dual mNGS results** | | | | |
| --- | --- | --- | --- | --- | --- | --- |
|  |  | **Definite**  **(reads)**  **(n=49)** | **Probable**  **(reads)**  **(n=22)** | **Possible**  **(reads)**  **(n=17)** | **Unlikely**  **(reads)**  **(n=9)** | **Indeterminate**  **(reads)**  **(n=0)** |
| 1 | *Klebsiella pneumoniae* | *Klebsiella pneumoniae*  (224) | N | N | N | N |
| 2 | *Klebsiella pneumoniae* | *Klebsiella pneumoniae*  (10) | N | N | N | N |
| 3 | *Candida tropicalis* | *Candida tropicalis*  (672) | N | N | N | N |
| 4 | *Escherichia coli* | *Escherichia coli*  (37) | *Pseudomonas aeruginosa*  (10) | N | N | N |
| 5 | *Pseudomonas aeruginosa* | *Pseudomonas aeruginosa*  (121) | N | N | N | N |
| 6 | *Klebsiella pneumoniae* | *Klebsiella pneumoniae*  (1870) | N | N | N | N |
| 7 | *Pseudomonas aeruginosa* | *Pseudomonas aeruginosa*  (1072) | N | N | N | N |
| 8 | *Escherichia coli* | *Escherichia coli*  (103) | N | N | N | N |
| 9 | *Klebsiella pneumoniae* | *Klebsiella pneumoniae*  (644) | *Staphylococcus hominis*  (30) | N | N | N |
| 10 | *Pseudomonas aeruginosa* | *Pseudomonas aeruginosa*  (37) | N | N | N | N |
| 11 | *Klebsiella pneumoniae* | *Klebsiella pneumoniae*  (705) | N | N | N | N |
| 12 | *Pseudomonas aeruginosa* | *Pseudomonas aeruginosa*  (239) | N | N | N | N |
| 13 | *Streptococcus mitis* | *Streptococcus mitis*  (25) | *Streptococcus pneumoniae*  (16) | N | N | N |
| 14 | *Pseudomonas aeruginosa* | *Pseudomonas aeruginosa*  (531) | N | N | N | N |
| 15 | *Pseudomonas aeruginosa* | *Pseudomonas aeruginosa*  (1705) | N | N | N | N |
| 16 | *Enterobacter aerogenes* | *Enterobacter aerogenes*  (110) | *Klebsiella pneumoniae*  (89) | N | N | N |
| 17 | *Serratia*  *marcescens* | *Serratia*  *Marcescens*  (91) | N | N | N | N |
| 18 | *Klebsiella pneumoniae* | *Klebsiella pneumoniae*  (346) | N | N | N | N |
| 19 | *Enterobacter cloacae* | *Enterobacter cloacae*  (794) | N | N | *Fusarium Verticillioide*  (8) | N |
| 20 | *Enterobacter cloacae* | *Enterobacter cloacae*  (31) | N | N | *Staphylococcus capitis*  (29) | N |
| 21 | *Escherichia coli* | *Escherichia coli*  (94) | N | N | N | N |
| 22 | *Pseudomonas aeruginosa* | *Pseudomonas aeruginosa*  (321) | N | N | N | N |
| 23 | *Streptococcus mitis* | *Streptococcus mitis*  (8) | N | N | N | N |
| 24 | *Stenotrophomonas maltophilia* | *Stenotrophomonas maltophilia*  (1524) | *Moraxella osloensis*  (389) | N | N | N |
| 25 | *Streptococcus pneumoniae* | *Streptococcus pneumoniae*  (11) | N | *Staphylococcus capitis*  (187) | N | N |
| 26 | *Aeromonas caviae* | *Aeromonas caviae*  (130) | N | *Staphylococcus xylosus*  (612) | N | N |
| 27 | *Escherichia coli* | *Escherichia coli*  (238) | N | N | N | N |
| 28 | *Klebsiella pneumoniae* | *Klebsiella pneumoniae*  (305) | N | N | N | N |
| 29 | *Klebsiella pneumoniae* | *Klebsiella pneumoniae*  (12) | N | *Lautropia mirabilis*  (47) | N | N |
| 30 | *Klebsiella pneumoniae* | *Klebsiella pneumoniae*  (13204) | N | *Staphylococcus haemolyticus*  (11) | N | N |
| 31 | *Candida tropicalis* | *Candida tropicalis*  (36) | N | N | N | N |
| 32 | *Staphylococcus hominis* | *Staphylococcus hominis*  (41) | *Enterococcus faecium*  (32) | N | N | N |
| 33 | *Staphylococcus epidermidis* | *Staphylococcus epidermidis*  (130) | N | N | N | N |
| 34 | *Streptococcus salivarius* | *Streptococcus salivarius*  (10) | N | N | N | N |
| 35 | *Klebsiella oxytoca* | *Klebsiella oxytoca*  (730) | *Pseudomonas aeruginosa*  (20) | N | N | N |
| 36 | *Acinetobacter baumannii* | *Acinetobacter baumannii*  (94) | *Stenotrophomonasmaltophilia*  (415) | N | N | N |
| 37 | *Stenotrophomonas maltophilia* | *Stenotrophomonas maltophilia*  (17171) | N | N | N | N |
| 38 | *Streptococcus viridan* | *Streptococcus viridan*  (16) | N | N | N | N |
| 39 | *Escherichia coli* | *Escherichia coli*  (22092) | *Enterococcus avis*  (30) | N | N | N |
| 40 | *Klebsiella pneumoniae* | *Klebsiella pneumoniae*  (165) | *Pseudomonas aeruginosa*  (24) | N | N | N |
| 41 | *Enterobacter cloacae* | *Enterobacter cloacae*  (10) | N | N | N | N |
| 42 | *Escherichia coli* | *Escherichia coli*  (112) | N | N | N | N |
| 43 | *Klebsiella pneumoniae* | *Klebsiella pneumoniae*  (16) | *Enterobacter cloacae*  (14) | N | N | N |
| 44 | *Enterobacter cloacae* | *Enterobacter cloacae*  (57) | N | *Candida sake*  (33) | N | N |
| 45 | *Enterobacter cloacae* | *Enterobacter cloacae*  (10) | N | N | N | N |
| 46 | *Escherichia coli* | *Escherichia coli*  (68) | N | N | *Staphylococcus equorum*  (146) | N |
| 47 | *Pseudomonas aeruginosa* | *Pseudomonas aeruginosa*  (252) | N | N | N | N |
| 48 | *Pseudomonas aeruginosa* | *Pseudomonas aeruginosa*  (248) | N | N | N | N |
| 49 | *Pseudomonas aeruginosa* | *Pseudomonas aeruginosa*  (35) | N | N | N | N |
| 50 | *Staphylococcus epidermidis* | N | N | N | *Propionibacterium humerusii*  (687) | N |
| 51 | *Streptococcus gordonii* | N | N | N | N | N |
| 52 | *Enterobacter cloacae* | N | N | *Klebsiella pneumoniae*  (12) | N | N |
| 53 | *Klebsiella oxytoca* | N | *Pseudomonas aeruginosa*  (10) | N | N | N |
| 54 | *Staphylococcus hominis* | N | *Klebsiella pneumoniae*  (99);  *Stenotrophomonas maltophilia*  (86) | N | N | N |
| 55 | *Escherichia coli* | N | N | *Leuconostoc mesenteroides*  (13) | N | N |
| 56 | *Pseudomonas aeruginosa* | N | N | *Moraxella nonliquefaciens*  (10) | N | N |
| 57 | *Proteus mirabilis* | N | N | N | N | N |
| 58 | *Escherichia coli* | N | N | *Ochrobactrum anthropi*  (15) | N | N |
| 59 | *Pseudomonas aeruginosa* | N | *Staphylococcus hominis*  (393) | N | N | N |
| 60 | *Escherichia coli* | N | *Acinetobacter baumannii*  (64) | *Staphylococcus coriolis*  (204) | N | N |
| 61 | *Pseudomonas aeruginosa* | N | *Staphylococcus hominis*  (4271) | *Candida parasmooth*  (8) | N | N |
| 62 | *Escherichia coli* | N | N | *Klebsiella pneumoniae*  (10)  *Acinetobacter ursingii*  (83) | N | N |

^a^ Numbers in brackets represent RPTM.

**Supplementary Table S5.** **The potential impact of mNGS results for antimicrobial management.**

| Aspect | Clinical impact^a^ | Case number, n (%)  N=300 |
| --- | --- | --- |
| The initial antimicrobial treatment  (0-48h) | Definitely effective | 57(19.0) |
|  | Probable effective | 74(24.7) |
|  | Likely effective | 19(6.3) |
|  | Not effective | 46(15.3) |
|  | Not applicable^b^ | 104(34.7) |
| The follow-up antimicrobial treatment  (after 48h) | Definitely effective | 66(22.0) |
|  | Probable effective | 77(25.7) |
|  | Likely effective | 17(5.7) |
|  | Not effective | 36(12.0) |
|  | Not applicable | 104(34.6) |
| Proposal of real-time availability of mNGS results on antimicrobial treatment | 1. Antibiotics need to be adjusted to cover the mNGS positive results that were not covered at the first fever, or replaced more sensitive antibiotics. | 89(29.7) |
|  | 2. Antibiotics need to be escalated according to the positive result of mNGS. | 6(2.0) |
|  | 3. Antibiotics need to be de-escalated or reduced according to the result of mNGS. | 62(20.6) |
|  | 4. The antibiotics had already covered the positive results of mNGS, and the experts panel had judged that the antibiotic treatment was effective. | 92(30.7) |
|  | 5. The negative mNGS result, or the result of mNGS was judged to be unlikely or indeterminate, and the experts panel had judged that antibiotics need not to be degraded or reduced. | 50(16.7) |
|  | 6. The patient was dead when the mNGS report become available. | 1(0.3) |
| Abbreviation: mNGS, metagenomics next-generation sequencing  ^a^ The effectiveness was judged according to the literature on antimicrobial susceptibility of the microorganism under consideration. | | |
